# Comparison of traditional and novel tip-flexible suctioning ureteral access sheath combined with flexible ureteroscope to treat unilateral renal calculi

**DOI:** 10.1007/s00345-023-04648-w

**Published:** 2023-10-11

**Authors:** Zhaolin Zhang, Tianpeng Xie, Fangzhi Li, Xiaoning Wang, Folin Liu, Bo Jiang, Xiaofeng Zou, Guoxi Zhang, Yuanhu Yuan, Rihai Xiao, Gengqing Wu, Biao Qian

**Affiliations:** 1 Department of Urology, First Affiliated Hospital of Gannan Medical University, No. 128, Jinling Road, Ganzhou, 341000 Jiangxi China; 2 Jiangxi Stone Prevention Engineering Technology Research Center, Ganzhou, 341000 Jiang Xi China; 3 First Clinical Medical College, Gannan Medical University, Ganzhou, 341000 Jiang Xi China

**Keywords:** Renal calculi, Retrograde intrarenal surgery, Ureteral access sheath, Flexible ureteroscopy

## Abstract

**Objectives:**

To compare the safety and efficacy of novel tip-flexible suctioning ureteral access sheath (NTFS-UAS) and traditional ureteral access sheath (T-UAS) combined with flexible ureteroscope for treating unilateral renal calculi.

**Materials and methods:**

The clinical data of 214 patients with unilateral renal calculi treated by NTFS-UAS (*n* = 102) and T-UAS (*n* = 112) combined with flexible ureteroscope from August 2021 to April 2022 were analyzed retrospectively. Demographic characteristics, stone-related parameters, operative time, stone-free rates (SFR), hospitalization time and complication rate (CR) were analyzed.

**Result:**

No significant difference was observed between the two groups in terms of demographic characteristics, stone-related parameters, intraoperative CR, and hospitalization time. The operative time of NTFS-UAS group was significantly shorter than T-UAS group (55.25 ± 11.42 min vs. 59.36 ± 15.59 min; *P* = 0.028). The NTFS-UAS group obtained significantly higher SFR on 1 day postoperatively (86.3% vs. 75.0%; *P* = 0.038), and higher SFR on 30 days postoperatively than T-UAS group (91.2% vs. 81.3%; *P* = 0.037). The hemoglobin loss of NTFS-UAS group (− 0.54 ± 0.69 g/dl) was significantly lower than T-UAS group (− 0.83 ± 0.66 g/dl; *P* = 0.002). There was a significantly lower incidence of overall CR (11.8% vs. 22.3%; *P* = 0.041), and infectious CR (8.8% vs. 18.8%; *P* = 0.037) in the NTFS-UAS group.

**Conclusion:**

Compared to T-UAS combined with flexible ureteroscope for treating unilateral renal calculi, NTFS-UAS had superiority in higher SFR on 1 day and 30 days postoperatively. Shorter operation time, lower hemoglobin loss, lower incidences of overall and infectious CR were observed in NTFS-UAS group.

**Registration number and date:**

ChiCTR2300070210; April 5, 2023.

**Supplementary Information:**

The online version contains supplementary material available at 10.1007/s00345-023-04648-w.

## Introduction

Urinary calculus is a worldwide urological disease, with a prevalence ranging from 1% to 13% in different regions [1]. The prevalence rate of renal calculi in China is 5.8%, including 5.1% in females and 6.5% in males [2]. Currently, the main therapeutic methods beyond conservative treatment for renal calculi include extracorporeal shock wave lithotripsy (ESWL) and minimally invasive endoscopic surgical methods, including percutaneous nephrolithotomy (PCNL) and retrograde intrarenal surgery (RIRS). Treatment plans depend on the characteristics of calculi, patient factors, surgeon experience and the condition of medical centers.

According to the guidelines of American Urologic Association (AUA) [3] and European Association of Urology (EAU) [4], patients with a burden of less than 20 mm in kidney calculi can choose RIRS as the first-line surgical treatment with good stone-free rate (SFR). The application of RIRS for urinary stones has increased significantly, and the indications have expanded due to developments in minimally invasive technology and equipment [5]. With the development of stone retrieval devices and miniaturized flexible ureteroscopes, RIRS is more widely used for treating renal calculi, even for high burden stones [6, 7]. The application of ureteral access sheath (UAS) in RIRS can improve surgical vision [8], reduce intrarenal pressure (IRP) [8, 9], and decrease postoperative infectious complications [10]. Several reports have demonstrated the superiority of suctioning UAS, including shorter operation time, higher SFR and lower incidence of infectious complications compared with traditional ureteral access sheath (T-UAS) [11–13], but none of these suctioning UAS can reach the renal calyces. A novel tip-flexible suctioning ureteral access sheath (NTFS-UAS) with flexible terminal was designed, which delivered the tip of the UAS to renal calyces. However, data comparing NTFS-UAS and T-UAS is lacking in RIRS. Therefore, we designed a retrospective controlled analysis to compare the efficacy and safety of NTFS-UAS and T-UAS combined with flexible ureteroscope (FURS) in treating unilateral renal calculi.

## Materials and methods

### Patients

Medical records of patients who successfully underwent RIRS with NTFS-UAS or T-UAS by the same surgeon in the First Affiliated Hospital of Gannan Medical University between August 2021 and April 2022 were retrospectively identified. Patients aged 18–70 years old with unilateral renal calculi were recruited, and patients with bilateral renal calculi who did not undergo the same session were also included. The exclusion criteria were as follows: (a) combined ipsilateral ureteral stone or contralateral upper urinary stone or lower urinary stone requiring simultaneous surgery; (b) any relative or absolute contraindications to RIRS; (c) presence of urinary anatomical abnormalities; and (d) previous urinary diversion surgery. In total, 102 patients undergoing surgery with NTFS-UAS and 112 patients with T-UAS were included. All patients underwent preoperative urinary non-contrast computed tomography (CT). For patients with normal renal function, intravenous urography (IVU) was recommended. The stone size was defined as the maximum diameter measured by CT. For multiple stones, the size was the sum of the maximum diameter of all stones. Stone hardness was defined as the average CT value. Preoperative antibiotics were administered according to urine analysis and midstream urine culture. Patients with positive urine culture were treated with antibiotics according to the sensitivity tests until the urine culture became negative. Patients with negative urine culture but with positive analysis for leukocytes and/or nitrites were treated with antibiotics according to local antimicrobial susceptibility for at least 3 days. Patients with negative urine culture and urine analysis were received a single dose of prophylactic antibiotics 1 h preoperatively.

## Surgical techniques

### T-UAS group

After satisfactory general anesthesia, the lithotomy position was applied for all patients. Under the guidance of a hydrophilic 0.035-inch guide wire, an 8/9.8 Fr semi-rigid ureteroscope was placed into the ureter and retrograded to the renal pelvis. The ureteroscope was then withdrawn, and the guide wire was left in the upper urinary tract. Next a 12/14 Fr T-UAS (Shenzhen Kang Yi Bo Technology Development Co., Ltd, Shenzhen, China) was inserted into the upper ureter. The length of the T-UAS was 45 cm for male patients and 35 cm for female patients. A digital FURS (Guangzhou Red Pine Medical Instrument Co., Ltd, Guangzhou, China) was inserted via the T-UAS, and then the end of the T-UAS was adjusted at ureteropelvic junction (UPJ) under its direct vision. After comprehensive inspection of the mucosa of renal collecting system, the proximal ureter and renal stones under perfusion flow were set to 60–100 ml/min. Then, 200 μm laser fiber was inserted through the working channel of the FURS for lithotripsy, and a holmium:yttrium aluminum garnet (Ho:YAG) laser was applied to pulverize calculi by interchangeably setting different parameters. Higher energy (0.6–1.2 J) and lower frequency (5–20 Hz) were set for fragmentation, and the dusting mode using low energy setting (0.2–0.6 J) and high frequency (20–30 Hz). For some calyceal calculi and stone fragments, a nitinol stone basket was applied to retrieve or relocate fragments when necessary. After all renal stones were lithotripsy to satisfactory fragments, a second comprehensive inspection of the renal collecting system and ureter was performed when removing the FURS and the T-UAS, a 5 F double-J (DJ) stent was inserted routinely.

### NTFS-UAS group

**Fig.1:**
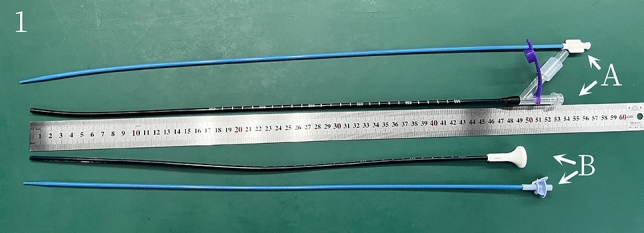
Full view of NTFS-UAS and T-UAS. **A** main body and obturator of NTFS-UAS.** B** main body and obturator of T-UAS

The NTFS-UAS and obturator (Zhangjiagang Huamei Medical Equipment Co., LTD, Zhangjiagang, China; Fig. 1) are both covered with hydrophilic lubricating coatings at outer surface, which facilitates passage through the urethra and ureter. The length of the NTFS-UAS is 45 cm for males and 35 cm for females, and it has the same luminal diameter, with an inside diameter of 12 Fr and an outside diameter of 14 Fr. The 10 cm flexible tip is located at the front end of the NTFS-UAS for rotation. The back end of the NTFS-UAS has a main straight tube and a 45-degree oblique tube. The main tube is closed by a rubber seal with a center aperture as the flexible ureteroscope pathway. The oblique tube with a pressure-regulating venting slit along the longitudinal axis acts as a suctioning channel connected to a vacuum device. The obturator is used for ureteral dilatation and UAS insertion.

**Fig.2:**
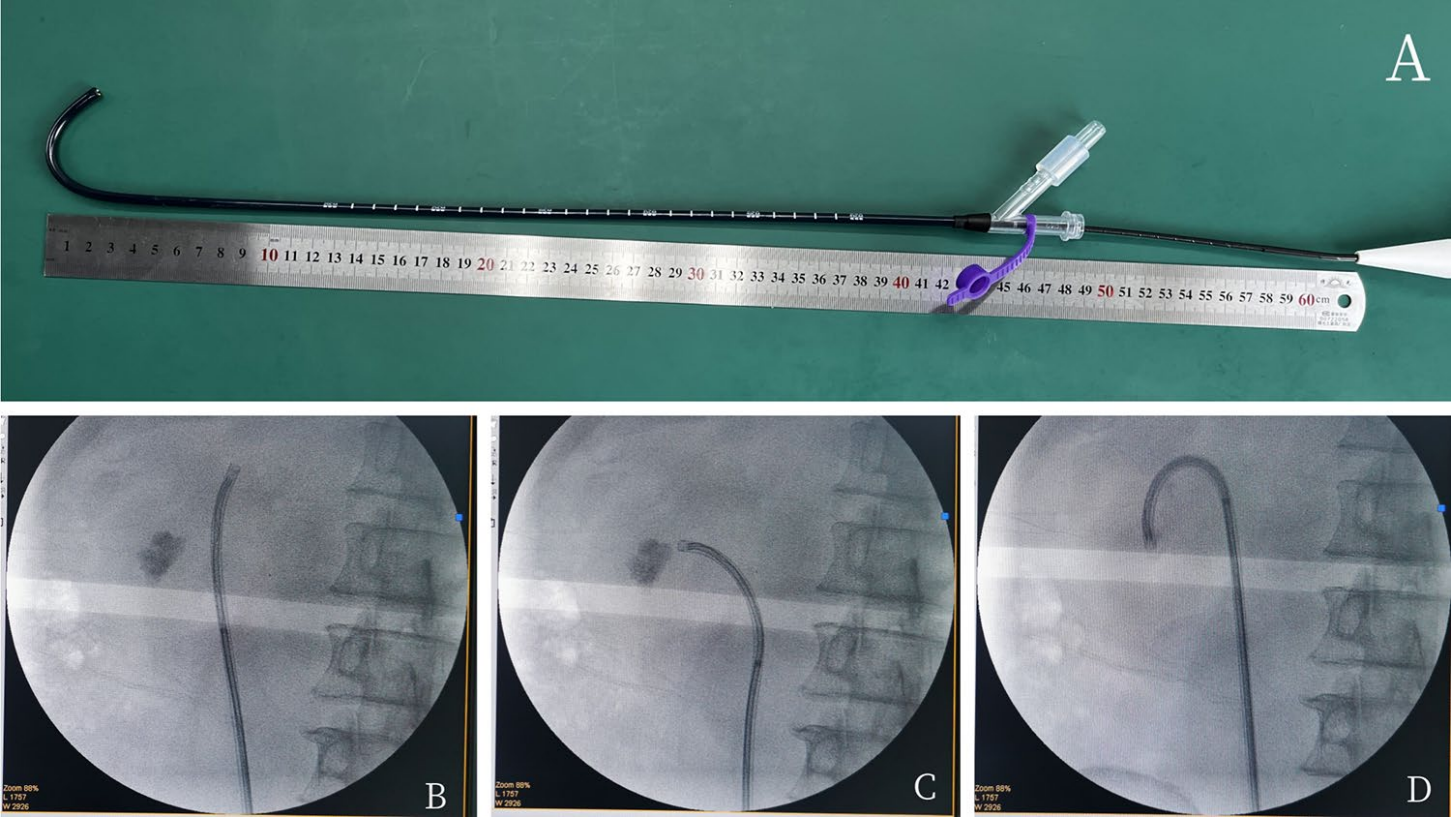
**A** Flexible tip of NTFS-UAS was bend assisted by flexible ureteroscope. **B** Located at upper renal calyx. **C** Located at middle renal calyx. **D** Located at lower renal calyx

**Fig.3:**
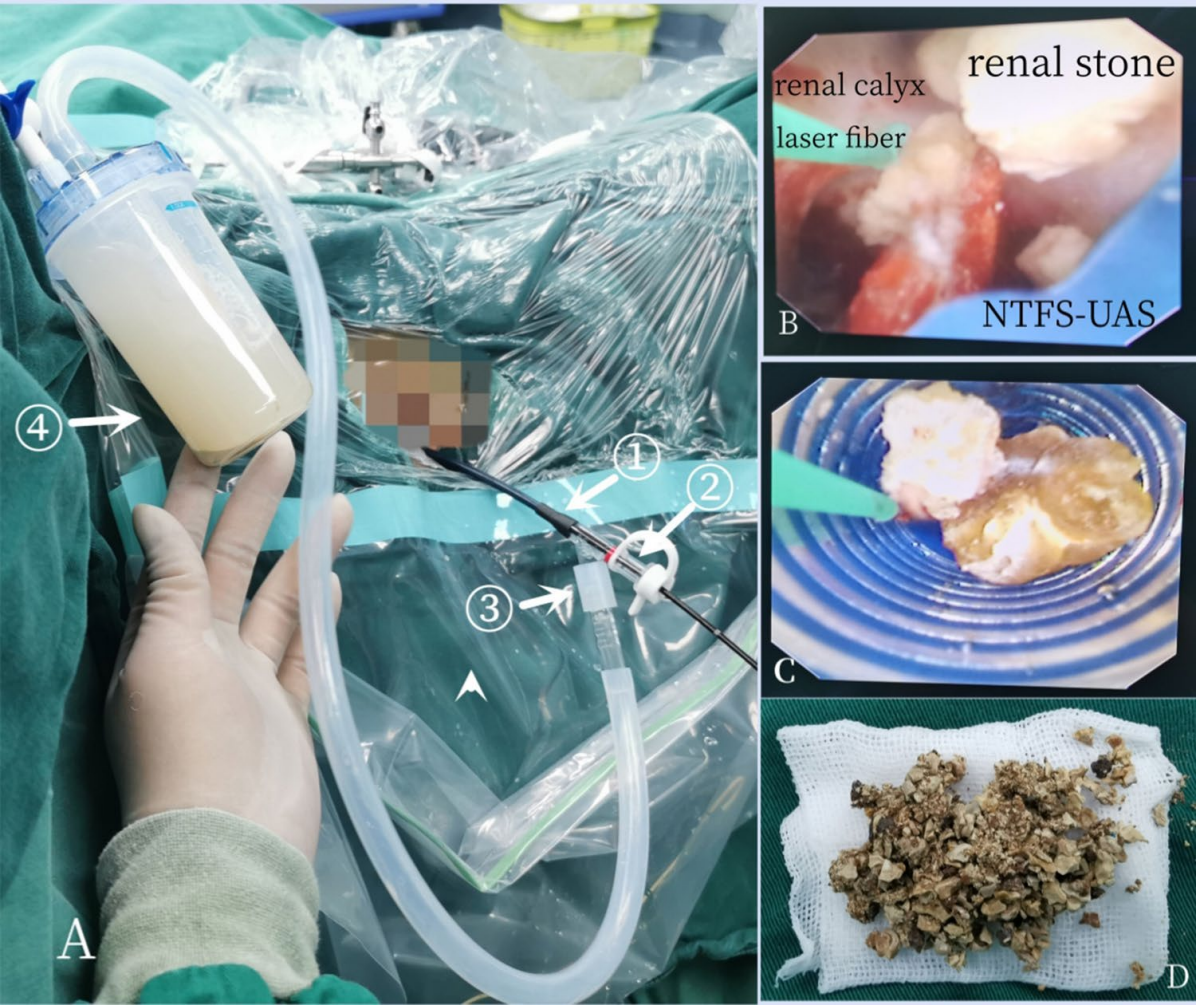
**A** The NTFS-UAS combined with flexible ureteroscope and vacuum device during operation: ①—main body of NTFS-UAS; ②—straight tube acted as flexible ureteroscope pathway; ③—oblique tube acted as suctioning channel connected with vacuum device and collecting bottle; ④—bottle for collecting fragments. **B** The tip of NTFS-UAS reached target calyx and stone. **C** Fragments fell out as the endoscope retreated. **D** Stone fragments

The patient position, anesthesia procedure, guide wire insertion, application of semi-rigid 8/9.8 Fr ureteroscope, placement of 12/14 Fr NTFS-UAS, and application of flexible ureteroscope were the same as those in the T-UAS group. After confirmation that the tip of the NTFS-UAS was located at the UPJ, the suctioning channel was connected to the vacuum device for an appropriate suctioning effect adjusted by a pressure-regulating venting slit, and the suctioning parameter of the vacuum device with negative pressure was set at -20 to -60 kPa. After comprehensive inspection of the mucosa of renal collecting system, FURS retreated to the opening of the NTFS-UAS. The flexible tip of NTFS-UAS could be bend assisted by the bendable tip of the FURS to rotate in the same direction. Under the surveillance of FURS, the NTFS-UAS was pushed or retreated to target position, including renal pelvis, calyces and target stones, especially the low renal pole (Fig. 2).The perfusion flow pattern, laser type and lithotripsy parameters, fiber type and nitinol basket were the same as those in T-UAS group, but the perfusion flow parameter was adjusted at a level of 60 ml/min to 140 ml/min. Stone segments could be removed from kidney through NTFS-UAS by circulation of irrigation flow and suctioning effect. The dust or tiny fragments could be suctioned toward the gap between the endoscope and the sheath and would fall out of the sheath. For fragments larger than gap but smaller than the internal diameter of UAS, the negative pressure and irrigation flow increased, and the fragments fell out as the endoscope retreated to the junction of the suction channel. After all renal stones were pulverized to satisfactory fragments and removed satisfactorily, the perfusion flow was decreased, suctioning was stopped, a guide wire was inserted. A second comprehensive inspection of renal collecting system and ureter was performed when removing the FURS and the NTFS-UAS. Routinely, a 5 F DJ stent was inserted. Finally, the stone fragments were collected in the suctioning bottle (Fig. 3).

Kidney–ureter–bladder (KUB) graphy and/or urinary CT were performed in both groups at 1 day and 1 month postoperatively. Stone-free status was considered as no residual stone or radiological residue fragment < 2 mm. The DJ stent was taken out regularly at 1 month postoperatively. Patients with ureteral stenosis or residual stones requiring a second procedure were treated 1 month after surgery. Routine follow-up was arranged for ultrasonography and/or KUB and/or non-contrast CT examination at 3 months after surgery.

### Study variables and statistical analysis

The preoperative demographic characteristics and baseline data of patients in the two groups were collected, including age, gender, comorbidities, American Society of Anesthesiologists (ASA) score, body mass index (BMI), surgical side, ipsilateral surgical history, midstream urine culture results, stone parameters, hydronephrosis and preoperative ureteral stent placement. The operation time, SFR, blood loss, stone composition, complications and hospitalization after operation were compared between the two groups. The operation time was calculated from endoscope insertion into the urethra to its removal. The amount of blood loss was assessed by measuring the variation in hemoglobin levels before and 24 h after operation. Intraoperative and postoperative complications were evaluated by the Satava classification [14] and Clavien‒Dindo classification [15], respectively.

Statistical analysis was executed using SPSS 26.0 Statistics (IBM, Armonk, NY). Continuous quantitative variables conforming to a normal distribution were reported as mean and standard deviation, the Satterthwaite *t*’ test was used for those with unequal variances and Student’s *t* test was used for groups with homogeneity of variance. For abnormally distribution continuous quantitative variables, the Wilcoxon signed-rank test was used. Qualitative variables were expressed as percentages (%) or numbers (n), according to total number and theoretical number, Chi-squared or Fisher’s exact probability test was used. Two-sided *P* -values less than 0.05 were considered indicators of statistical significance.

## Results

The demographic characteristics and baseline data (gender, age, BMI, ASA score, comorbidities, operative side, surgical history of operative side), stone size, stone hardness and stone location, hydronephrosis, proportion of positive midstream urine culture and preoperative ureteral stent placement showed no significant difference between the two groups (Table 1).

**Table 1:** Demographic characteristics and baseline data of two groups

	NTFS-UAS group	T-UAS group	*t/χ*^*2*^	*P* value
Total number, (*n*)	102	112		
Age (years), mean ± SD	47.69 ± 9.18	46.75 ± 11.87	0.648	0.517
Gender, *n* (%)			1.978	0.160
Male	55 (53.9%)	71 (63.4%)		
Female	47 (46.1%)	41 (36.6%)		
BMI (kg/m^2^), mean ± SD	24.25 ± 2.97	23.54 ± 3.37	1.645	0.101
ASA score, *n* (%)			0.987	0.667
I	12 (11.8%)	9 (8.0%)		
II	88 (86.3%)	101 (90.2%)		
III	2 (1.9%)	2 (1.8%)		
Comorbidities, *n* (%)			1.841	0.616
Hypertension	18 (17.6%)	19 (17.0%)		
Diabetes mellitus	8 (7.8%)	5 (4.5%)		
Renal insufficiency	10 (9.8%)	8 (7.1%)		
Operative side, *n* (%)			0.956	0.328
Left	46 (45.1%)	58 (51.8%)		
Right	56 (54.9%)	54 (48.2%)		
History of surgery on ipsilateral side, *n* (%)			1.256	0.764
RIRS	9 (8.8%)	13 (11.6%)		
PCNL	8 (7.8%)	12 (10.7%)		
Open surgery	3 (2.9%)	4 (3.6%)		
Midstream urine culture, *n* (%)			1.843	0.175
Positive	26 (25.5%)	20 (17.9%)		
Negative	76 (74.5%)	92 (82.1%)		
Renal stone size (range); mm, mean ± SD	18.47 ± 4.67	18.20 ± 4.46	0.440	0.661
Renal stone hardness (HU), mean ± SD	1061.67 ± 272.54	1096.38 ± 240.94	0.989	0.324
Stone location, *n* (%)			0.417	0.981
Pelvis	41 (40.2%)	43 (38.4%)		
Upper calyx	10 (9.8%)	9 (8.0%)		
Middle calyx	9 (8.8%)	11 (9.8%)		
Lower calyx	12 (11.8%)	15 (13.4%)		
Multiple calyxes	30 (29.4%)	34 (30.4%)		
Hydronephrosis, *n* (%)			3.530	0.314
No	27 (26.5%)	24 (21.4%)		
Mild	47 (46.1%)	57 (50.9%)		
Moderate	22 (21.6%)	29 (25.9%)		
Gross	6 (5.9%)	2 (1.8%)		
Preoperative ureteral stent placement, *n* (%)			0.213	0.644
Yes	46 (45.1%)	47 (42.0%)		
No	56 (54.9%)	65 (58.0%)		

*NTFS-UAS* novel tip-flexible suctioning ureteral access sheath, *T-UAS* traditional ureteral access sheath, *SD* standard deviation, *BMI* body mass index, *ASA* American Society of Anesthesiologists, *RIRS* retrograde intrarenal surgery, *PCNL* percutaneous nephrolithotomy, *HU* Hounsfield Unit

The operation time in the NTFS-UAS group (55.25 ± 11.42 min) was significantly shorter than that in T-UAS group (59.36 ± 15.59 min; *P* = 0.028). The hemoglobin loss was − 0.54 ± 0.69 g/dl in NTFS-UAS group and − 0.83 ± 0.66 g/dl in T-UAS group, with a significant difference (*P* = 0.002). Postoperative hospitalization did not differ between the two groups (2.86 ± 1.11 days vs. 2.76 ± 1.51 days; *P* = 0.570).

All patients received KUB imaging test on day 1 and day 30 after operation. CT was performed for patients with suspicious residual stone fragments based on KUB or unclear surgical view during the operation. Postoperative non-contrast CT was applied in 39.2% of patients in NTFS-UAS group and 33.9% in T-UAS group (*P* = 0.422). Compared with T-UAS group, NTFS-UAS group (86.3% vs. 75.0%; *P* = 0.038) showed a significantly higher SFR at postoperative day 1. The SFR at postoperative day 30 was significantly higher in NTFS-UAS group (91.2%) than in T-UAS group (81.3%; *P* = 0.037).

Intraoperative complications did not occur in either group. Compared with T-UAS group, the overall incidence of complications in NTFS-UAS group was significantly lower (11.8% vs. 22.3%; *P* = 0.041). In NTFS-UAS group, the incidence of infectious complications, including fever, urosepsis requiring only additional antibiotics and septic shock, was significantly lower (8.8% vs. 18.8%; *P* = 0.037). The incidence of persistent hematuria in the two groups was comparable. One patient in T-UAS group suffered from subcapsular hematoma and was cured with percutaneous nephrostomy. Two patients in T-UAS group suffered steinstrasse and were managed by ureteroscopic lithotripsy. There was 1 case of double-J stent displacement in each group, which was corrected by ureteroscopy under local anesthesia. Other complications, such as ureteral injury, ureteral rupture or tearing, severe bleeding, and acute renal failure, did not occur in either group.

For stone composition analysis, 85 patients in NTFS-UAS group and 92 patients in T-UAS group were tested, showing no significant difference (*P* = 0.777). Calcium oxalate was the predominant type. Table 2 summarizes the intraoperative and postoperative results.

**Table 2:** Clinical outcomes of NTFS-UAS group and T-UAS group

	NTFS-UAS group	T-UAS group	*t/χ*^*2*^	*P* value
Total number, (*n*)	102	112		
Operative time (min), mean ± SD	55.25 ± 11.42	59.36 ± 15.59	2.209	0.028
Hemoglobin loss (g/dl), mean ± SD	− 0.54 ± 0.69	− 0.83 ± 0.66	3.081	0.002
Postoperative hospitalization (days), mean ± SD	2.86 ± 1.11	2.76 ± 1.51	0.569	0.570
SFR at postoperative day 1, *n* (%)	88 (86.3%)	84 (75.0%)	4.302	0.038
SFR at postoperative day 30, *n* (%)	93 (91.2%)	91 (81.3%)	4.364	0.037
Total complications, Clavien grade classification, *n* (%)	12 (11.8%)	28 (25.0%)	6.153	0.013
Fever (> 38 °C) (G I)	4 (3.9%)	11 (9.8%)	2.851	0.091
Persistent hematuria (G I)	2 (2.0%)	3 (2.7%)	0.000	1.000
Urosepsis only need additional antibiotics (G II)	4 (3.9%)	7 (6.3%)	0.594	0.441
Subcupsular hematoma (G III)	0	1 (0.9%)	–	–
Steinstrasse (G III)	0	2 (1.8%)	–	–
Ureteroscopy under local anesthesia (G III)	1 (1.0%)	1 (0.9%)		1.000
Septic shock (G IV)	1 (1.0%)	3 (2.7%)	0.169	0.681
Stone composition, *n* (%)	85	92	1.901	0.777
Calcium oxalate	50 (58.8%)	55 (59.8%)		
Calcium phosphate	13 (15.3%)	17 (18.5%)		
Uric acid	9 (10.6%)	7 (7.6%)		
Struvite	12 (14.1%)	10 (10.9%)		
Cystine calculus	1 (1.2%)	3 (3.2%)		

*NTFS-UAS* novel tip-flexible suctioning ureteral access sheath, *T-UAS* traditional ureteral access sheath, *SD* standard deviation, *SFR* stone-free rates

## Discussion

RIRS is a minimally invasive procedure that has gained worldwide popularity for treating renal calculi, owing to its improved techniques and novel surgical instruments [16]. The application of UAS during RIRS can prevent high IRP, accelerate perfusion and drainage [17] and reduce infectious complications [18]. Modified suctioning UASs have been reported for treating urinary stones and have shown good outcomes [11–13]. However, to the authors' knowledge, there is a lack of data comparing NTFS-UAS and T-UAS combined with a digital flexible ureteroscope; therefore, this retrospective study was designed to confirm the safety and efficacy of NTFS-UAS.

The presence of UAS is associated with a decrease in IRP by facilitating the flow and drainage of irrigation fluid in the collecting system. Other important factors of IRP include UAS size, UAS/scope caliber ratio [19], gap between UAS and ureteroscope [20], location of UAS, occupied scope working channel, irrigation style and pressure [21] and vacuum-assisted ureteral access sheath [22]. Different from T-UAS located at ureteropelvic junction (UPJ), NTFS-UAS can be adjusted to renal pelvis and target renal calices as needed to provide sufficient irrigation-suctioning space, avoid blocking UAS opening by the mucous membrane of UPJ [22] and maintain low IRP. Combined with the vacuum device, NTFS-UAS enables a continuous irrigation-suctioning cycle, enhances the outflow of intrarenal fluid in a timely manner, and maintains a lower level of IRP.

Compared with T-UAS, NTFS-UAS has the following characteristics. First, the suctioning tube connected with the vacuum device keeps its IRP low during operation, allowing relatively larger irrigation flow and higher irrigation pressure during operation, keeping its surgical vision clearer and facilitating the aspiration of stone fragments and dust. Second, the flexible tip of NTFS-UAS can be adjusted to target calyces, especially for the lower renal pole, and deep and dilated calyces, differing from the suctioning UAS placed at UPJ reported by Zhu et al*.* [11]. According to the hydrodynamic effect theory of fluid and the vacuum cleaner effect in continuous-flow, stone fragments located within 10 mm in front of endoscope tip, near or at the opening of the sheath were most effectively suctioned towards the gap between endoscope and the sheath and fell out of the sheath [23]. For fragments larger than the gap but smaller than the internal diameter of UAS, increasing the negative pressure and irrigation flow is suggested, which results in the fragments falling out with progressive withdrawal of as the endoscope. The tip of NTFS-UAS can be bent to lower renal pole assisted with flexible ureteroscope for direct lithotripsy and suction. The space of deep and dilated calyces was narrowed after suction via NTFS-UAS, which was attributed to reducing stone movement and improving lithotripsy effectiveness. In addition, debris and dust were immediately suctioned, reducing the number of bacteria and the absorption of endotoxic substrates, resulting in a high SFR, a low incidence of infectious complications, and a reduces need for stone baskets or forceps. Furthermore, NTFS-UAS can be guided to target position under the direct view of flexible ureteroscope, which can reduce the damage to of ureter and the collecting system. Moreover, active control of negative pressure by adjusting width of the venting slit along the longitudinal axis of suctioning channel allows the surgeon to address different situations and avoid insufficient or excessive suction.

The SFR of NTFS-UAS group on day 1 (86.3%) and 30 days (91.2%) after operation were both significantly higher than those of patients with similar stone burdens who received T-UAS [10, 24]. RIRS typically removes fragments with a stone basket or forceps or pulverizes them into tiny fragments or dust that are actively expelled by the patient. However, the fragments or dust increased during lithotripsy performed with T-UAS, which led to poor surgical vision and may increase possibility of residual fragments that were too large to pass spontaneously [25]. The application of NTFS-UAS had a direct suction effect on fragments and dust, keeping the field view clear, reducing residual fragments, and completely lithotripsied all stones. Zhu et al*.* reported a retrospective controlled study of suctioning UAS and traditional UAS, and both involved location at the UPJ [11]. The SFR in the suctioning UAS group at postoperative day 1 was significantly higher than that in traditional UAS group (82.4% vs. 71.5%; *P* = 0.02), but the SFR in the two groups at 1 month after surgery was comparable (88.8% vs. 82.9%; *P* = 0.13) [11]. The SFRs at 1 day and 30 days postoperatively in the NTFS-UAS group in our study were higher than those reported by Zhu et al*.*, which may be related to the different locations of the UAS and the flexible tip design of NTFS-UAS. In Zhu’s report, the ureteral catheter was placed in a suctioning UAS, and then saline was injected through catheter into renal collecting system to form circulation to facilitate artificial aspiration and removal of larger fragments. However, we bent the tip of NTFS-UAS to capture the fragments in renal calices, and the fragments were directly suctioned out with the withdrawal of flexible ureteroscope. Zeng et al*.* reported that the overall immediate SFR of 74 patients who underwent modified suctioning UAS was 97.3%, which was higher than our results [13]. However, these patients were diagnosed with ureteral stones or steinstrasses, 67 patients underwent ureteroscopic lithotripsy, and only 7 patients underwent flexible ureteroscopic lithotripsy [13].

The operative time of NTFS-UAS group was significantly shorter than that of T-UAS group, which was similar to results reported by Zhu et al*.* [11]. In their study, a shorter operative time was obtained in the suctioning UAS group (49.7 ± 16.3 min) than in the T-UAS group (57.0 ± 14.0 min, *P* < 0.001). A retrospective matched-pair analysis conducted by Qian et al*.* showed comparable operative times between the suctioning and non-suctioning UAS groups, but the mean time was longer in the non-suctioning UAS group (80 min vs. 72.9 min) [18]. The difference in operative time was attributed to clearer surgical vision and better immediate clearance ability of NTFS-UAS. In addition, decreased re-entries of baskets or forceps could shorten the surgical time.

The overall CR and infectious CR in the T-UAS group were significantly higher than those in the NTFS-UAS group. Fever was the most common complication and septic shock was the most dangerous complication, but both were comparable between the two groups. Similarly, Zhu et al*.* indicated that the incidence of overall complications in the suctioning UAS group was significantly lower. Unlike the results in this study, the incidence of fever was significantly lower than that in the traditional UAS group [11]. Qian et al*.* reported a lower incidence of postoperative fever or systemic inflammatory response syndrome in suctioning UAS group [18]. Infectious complications after RIRS may be directly related to intrarenal pressure [26], and inappropriate irrigation or insufficient drainage may result in increased IRP, which may lead to renal damage, liquid reflux or extravasation, infection spread, urosepsis, or infectious shock [27]. The application of NTFS-UAS can deliver timely fluid suction in the renal collecting system to theoretically retain lower IRP, which may reduce the infectious complications of RIRS. However, our study lacked real-time IRP data to confirm the hypothesis. In addition to the fluid in the renal collecting system, infectious stone fragments, blood clots, suppurative floc, bacteria, bacterial endotoxin and abscess pus can also be suctioned through NTFS-UAS, which can reduce the absorbed infectious substances and infectious complications.

Our current study has several limitations. First, it is a retrospective study with limited samples, and potential patient selection bias cannot be eliminated. Second, although suctioning UAS could theoretically reduce IRP, a pressure feedback device for measuring real-time IRP was lacking in our study, so further validation is needed. Third, not all patients underwent CT, CT was performed for patients with suspicious residual stone fragments based on KUB or unclear surgical view during the operation. Some significant residual stone fragments may not be detected on KUB, and CT is superior to KUB. Due to these limitations, a stricter designed prospective randomized controlled study with large cases is suggested.

## Conclusion

According to our research results, compared with T-UAS combined with flexible ureteroscope for treating unilateral renal calculi, NTFS-UAS showed advantages of a higher SFR 1 day and 30 days postoperatively. NTFS-UAS possessed superiority of shorter operation time, lower hemoglobin loss, and lower incidences of overall CR and infectious CR.

### Supplementary Information

Below is the link to the electronic supplementary material.

Supplementary file1 (XLSX 27 KB)

## Data Availability

The original contributions presented in the study are included in the article/Supplementary Material, further inquiries can be directed to the corresponding author.
